# Open Reduction and Internal Fixation of a Pediatric Apophyseal Calcaneus Fracture Using Cannulated Screws: A Case Report

**DOI:** 10.7759/cureus.32341

**Published:** 2022-12-09

**Authors:** Dyrian Wandick, Smitha Mathew, Alisa Malyavko, Sean Tabaie

**Affiliations:** 1 Department of Orthopedic Surgery, Howard University Hospital, Washington, USA; 2 Department of Orthopedic Surgery, MedStar Union Memorial Hospital, Baltimore, USA; 3 Department of Orthopedic Surgery, George Washington University School of Medicine and Health Sciences, Washington, USA; 4 Department of Orthopedic Surgery, Children's National Hospital, Washington, USA

**Keywords:** hardware, cannulated screw, pediatric fractures, open reduction internal fixation, calcaneus fractures

## Abstract

We report a case of a 12-year-old boy who sustained a displaced calcaneal apophysis fracture, which was analogous to a bony avulsion of the insertion of the Achilles tendon, secondary to an awkward landing while jumping at a trampoline park. Treatment with open reduction and internal fixation with cannulated screws provided a novel approach to fixation for this type of fracture in the pediatric population.

## Introduction

Calcaneus fractures in children are rare injuries that are often missed radiographically. They usually occur due to a fall from height with an axial load on the heel. These injuries are often missed radiographically because of the small subtle abnormalities that may be seen with calcaneus fractures [1,2].

Calcaneus fractures in the pediatric population are classically described as displaced versus nondisplaced and intra-articular versus extra-articular. Unlike the adult population, most calcaneus fractures in children are thought to be consequences of low-energy trauma, usually extra-articular, and treated with a period of immobilization due to the remodeling potential of the immature calcaneus [3].

The calcaneus is the only tarsal bone that develops an epiphysis and secondary ossification center. Displaced fractures through the calcaneal apophysis can theoretically cause growth effects on the calcaneus and hindfoot. Fractures specific to the calcaneal apophysis have rarely been described [4]; however, it is thought that fractures through the apophysis can be described and treated like long-bone fractures that occur in the physeal and epiphyseal regions [5].

We report a case of a 12-year-old boy who presented with a displaced calcaneal apophyseal fracture and underwent open reduction internal fixation with a novel technique.

## Case presentation

**Figure 1:**
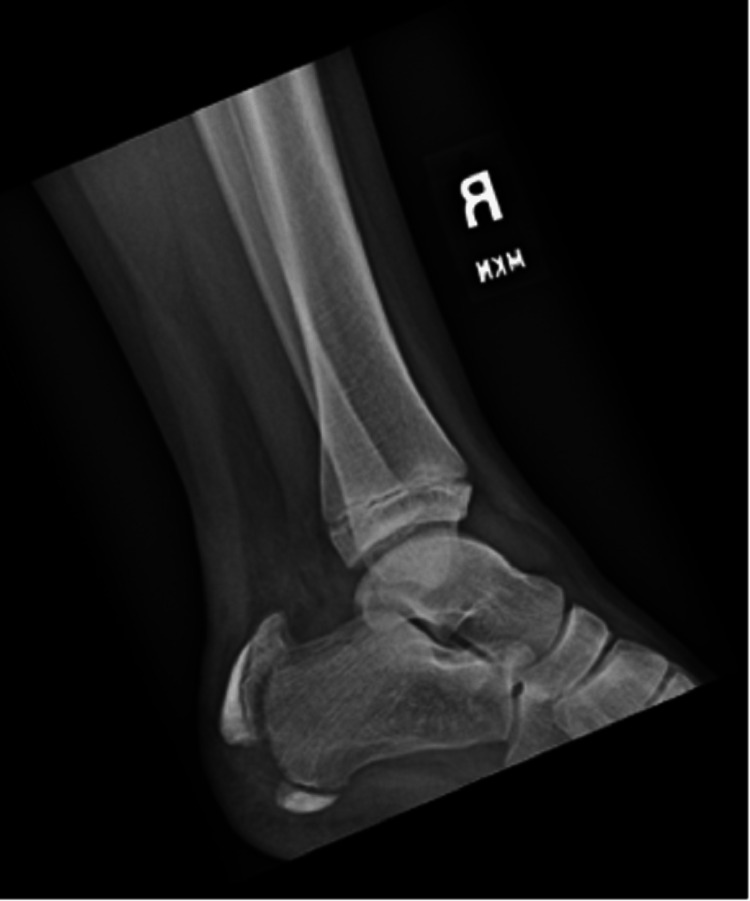
Plain Lateral radiograph of the right foot demonstrating a displaced fracture through the apophysis of the calcaneus with a cranial displacement of the proximal fracture fragment.

A 12-year-old boy with no past medical history presented with complaints of right heel pain. The patient reported that the day before, he was at a trampoline facility where he landed awkwardly on his right heel. He noticed immediate pain as well as swelling at the back of the foot. He initially presented to an urgent care facility where radiographs were obtained that showed a fracture through the apophysis of the calcaneus with cranial or superior displacement. He was placed in a posterior splint and told to follow up with orthopedics. Due to poor pain control, the patient presented to Children's National Medical Center. His physical exam was notable for ecchymosis and tenderness around the ankle and heel. His strength, sensation, and vascular exams were intact distal to the injury. A radiograph of the right foot is consistent, with a fracture through the calcaneal apophysis with a cranial displacement of the proximal fracture fragment (Figure 1).

Based on the patient's injury pattern, we recommended open reduction and internal fixation of his right displaced calcaneal apophysis fracture. All risks, benefits, and alternatives were discussed and outlined in the informed surgical consent. The patient's mother was in agreement, and surgical consent was signed.

The patient was taken to the operating room for general anesthesia. A full surgical time-out was performed. A prophylactic dose of IV antibiotics was given to the patient. The patient's right side was elevated on some blankets to put him in a sloppy lateral position. We then placed a bone foam under his right leg. A nonsterile, right-sided thigh tourniquet was placed. The right leg was prepped and draped in a typical sterile fashion.

**Figure 2:**
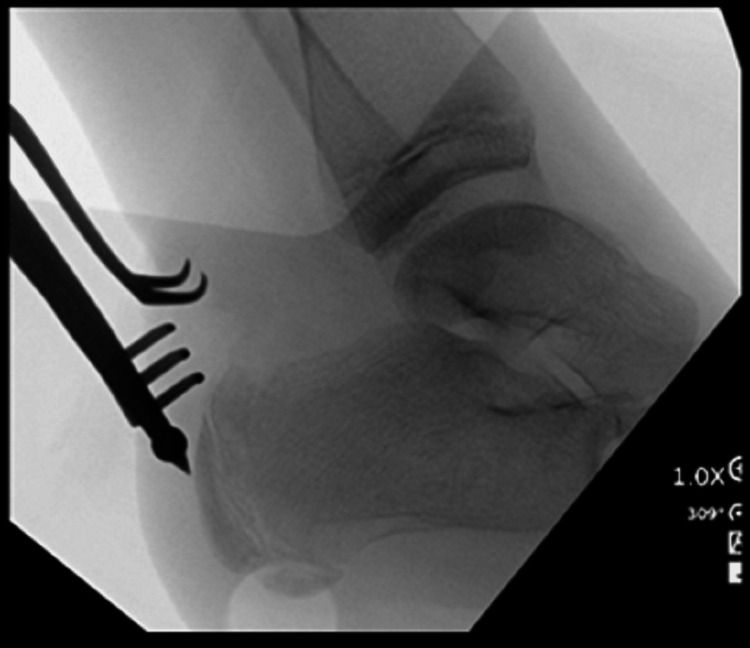
Displaced fracture fragment being reduced with a ball spike pusher to its anatomic location.

Fluoroscopy was then used to mark the location of the displaced fragment. An approximate 2 cm incision just medial to the Achilles tendon was made. Initial sharp dissection was carried out and then blunt dissection down to the fracture site. We used a freer to free up the displaced proximal fracture fragment and irrigated the fracture fragment to make him more mobile. This open approach to the Achilles also allowed us to assess the integrity of the tendon, and there was no traumatic or iatrogenic injury to the Achilles tendon. We then used a ball spike pusher and pushed on the fracture fragment to its anatomic location (Figure 2).

**Figure 3:**
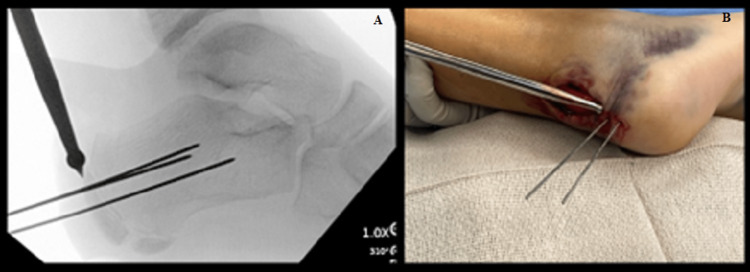
Provisional reduction being held with 2 K-Wires. (A) Lateral radiograph showing placement of two K-wires. (B) Image of the right heel showing two K-wires in place. K-wires, Kirschner wires

With fluoroscopy, we were able to visualize that the fracture fragment was reduced in a proper position. Biplanar fluoroscopic images were obtained in the lateral and Harris axial views. We then placed two appropriately sized guidewires within the fracture fragment into the calcaneus itself to hold it in place (Figure 3).

**Figure 4:**
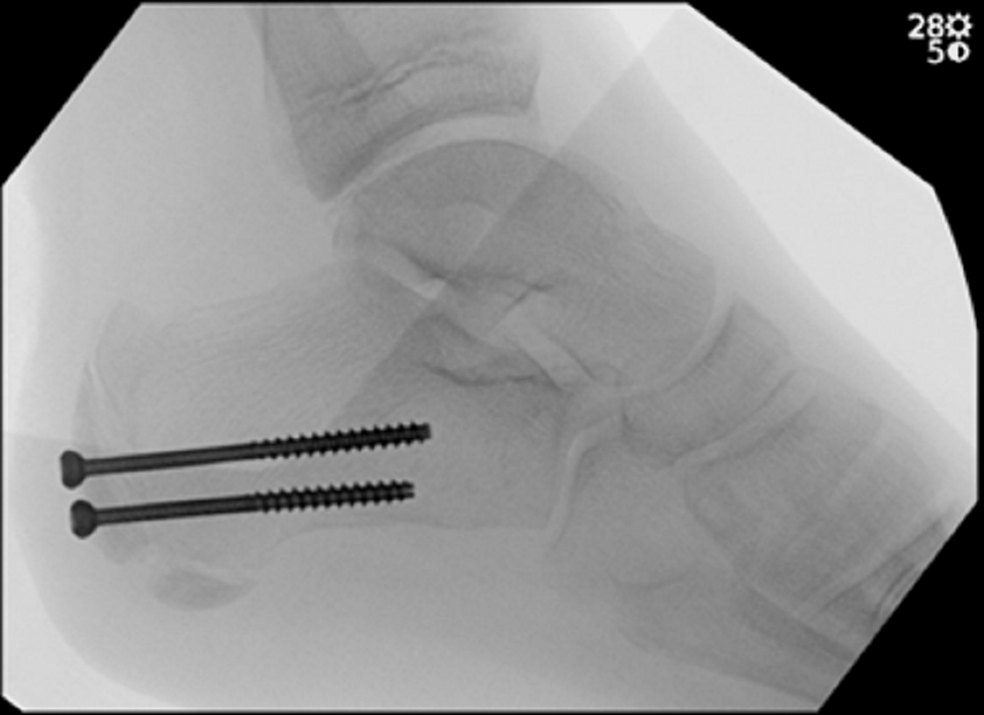
Cannulated screws holding the fracture fragment reduced.

We then proceeded to measure the length of the proposed screws. Once the screws were measured, we used a cannulated drill to drill out the near cortex. The appropriate length screws were then placed and had a good bite within the fracture fragment. We then took final fluoroscopic images showing the fracture was appropriately reduced and stable (Figure 4).

Finally, we performed the Thompson test to reassess the tension of the Achilles tendon. The tension of the Achilles tendon was appropriate. Final fluoroscopic images were saved to the patient's record. The wound was then irrigated copiously and closed in the usual fashion. The patient was placed into a well-padded, short-leg splint, with the foot and ankle slightly plantarflexed. He was made non-weight-bearing, with crutches in the affected extremity.

**Figure 5:**
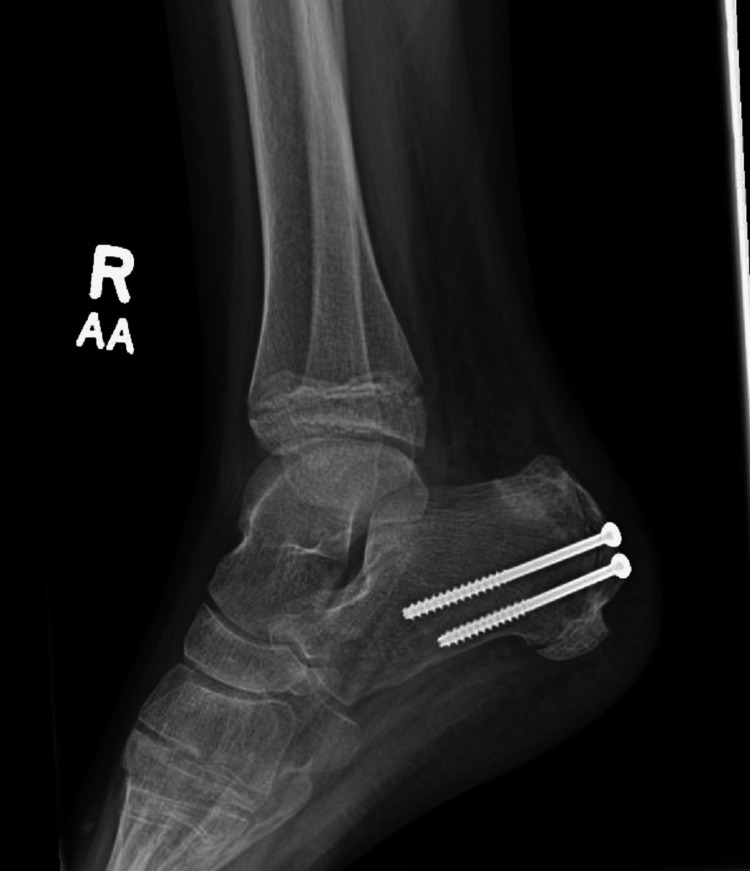
Lateral view of the right foot after fracture fixation.

**Figure 6:**
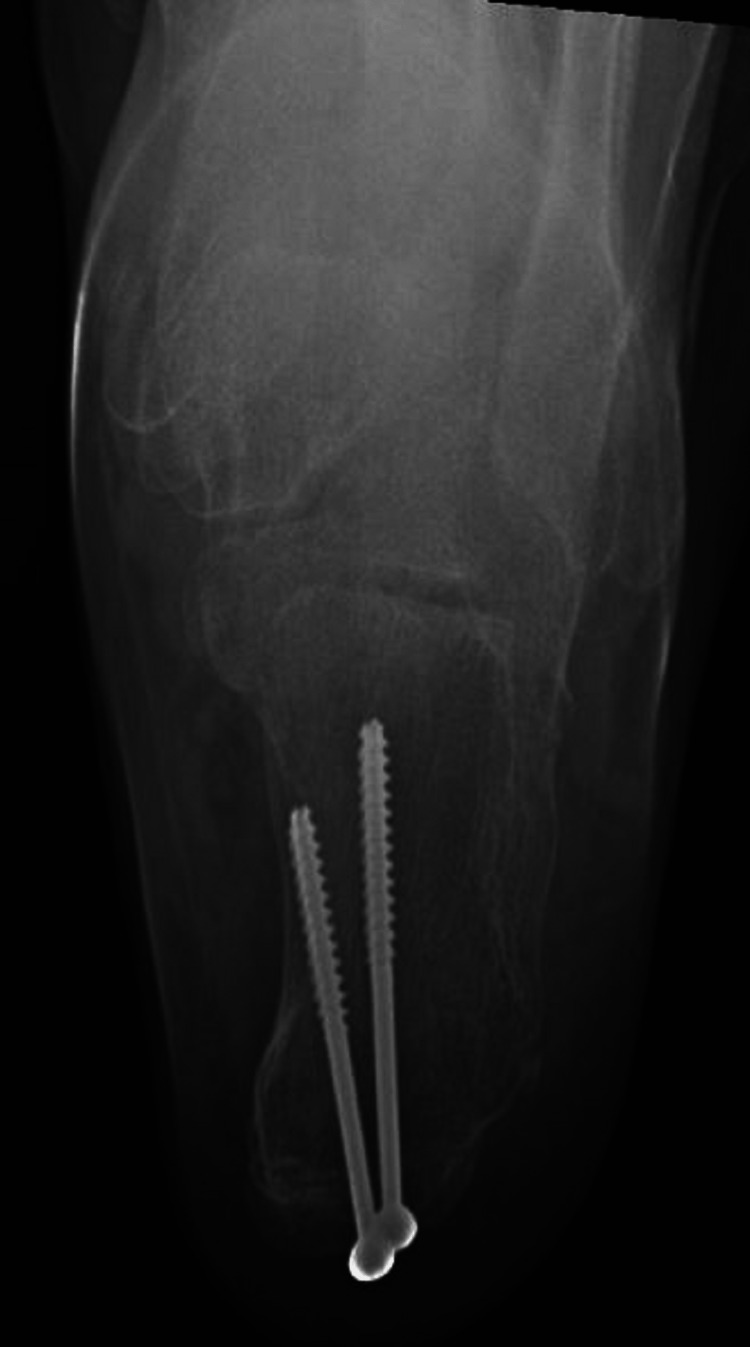
Axial view of the right foot after fracture fixation.

The patient presented approximately one week post-op for his first post-op appointment. The cast was removed. The incision was clean and dry, and the patient was neurovascularly intact distally. New radiographs were obtained that showed no significant interval change from intraoperative fluoroscopic images. The fracture was well reduced. Hardware was in place without any areas of concern (Figures 5-6). The patient was placed back in a short-leg cast with slight plantar flexion, and a checkup was scheduled four weeks post-op. 

 

The patient was seen two months post-op, and the cast boot was discontinued at that time. At the three months post-op appointment, the patient was able to bear weight and actively plantar and dorsiflex the ankle without any reported pain. The patient had intact light touch sensation and neurovascular function. Lateral and Harris axial radiographs showed a well-reduced fracture with hardware in place.

## Discussion

Calcaneus fractures among the pediatric population are rare, unlike the adult population. Calcaneal apophysis fractures are even less frequently reported and described in the literature. This case report presents a 12-year-old boy with a displaced calcaneal apophysis fracture. The fracture pattern of this patient’s injury is analogous to an avulsion fracture of the Achilles tendon. The patient successfully underwent open reduction internal fixation with a ball spike pusher used to reduce the fracture and two cannulated screws to hold the reduction in place, when he presented a day after the injury. This novel approach to fracture fixation was aimed to provide adequate and stable fracture fixation and anatomic reduction.

Walling et al. described pediatric calcaneal apophyseal avulsion fractures by the standard classification applied to growth plate injuries in long bones [5]. However, the fracture pattern reported in our patient does not fit Walling et al.’s proposed classification. Our fracture presented with a cranial displacement of the fracture fragment, resulting in a fracture that is equivalent to an Achilles tendon rupture. It is thought that this injury pattern occurs secondary to an excessive traction loading on the heel that in adults would result in an Achilles tendon rupture [4]. In pediatric patients, where the two centers of ossification of the apophysis are weaker than the tendon, this suspected force results in a fracture through the apophysis with the cranial displacement of the proximal fracture fragment.

Other recent case reports of calcaneal apophyseal fracture in the pediatric population have been reported. In these cases, the patient underwent open reduction and internal fixation using a combination of bioabsorbable suture tacks and pins, using the tension band wiring technique for fixation and a clip and plantarflexion for reduction [6,7]. One of the reported cases had hardware irritation, failure, and pin back out six months postoperatively. The patient was able to tolerate the removal of hardware with local anesthesia and went on to full healing with no complaints of pain at his two-year follow-up. Our proposed method of fixation with two cannulated screws aims to provide another alternative for stable anatomic fracture fixation for the rare injury of calcaneal apophyseal fractures.

Limitations of this study include a sample size of one and a relatively short follow-up period. The patient recovered and no longer came in for follow-up visits. This limited the ability to track the patient's progress, radiographs, and potential implant removal in the long term. 

## Conclusions

In conclusion, we report the successful use of open reduction internal fixation with cannulated screws in treating a displaced calcaneus fracture in the pediatric population. No perioperative or postoperative complications were encountered during the procedure, and the patient successfully recovered and returned to full functionality. Although fractures of the calcaneal apophysis are rare, they can result in stunted growth of the calcaneus due to disruption of the ossification center. For this reason, it is crucial to treat these fractures in a way that provides stable fracture fixation for proper healing. 
